# Effect of epilepsy on autism symptoms in Angelman syndrome

**DOI:** 10.1186/s13229-017-0185-1

**Published:** 2018-01-08

**Authors:** Kristin A. Bakke, Patricia Howlin, Lars Retterstøl, Øivind J. Kanavin, Arvid Heiberg, Terje Nærland

**Affiliations:** 1 NevSom, Department of Rare Disorders, Oslo University Hospital, Oslo, Norway; 2 Institute of Psychiatry, Psychology and Neuroscience, King’s College London, London, UK; 3 Faculty of Health Sciences, University of Sydney, Sydney, NSW Australia; 4 Department of Medical Genetics, Oslo University Hospital, Oslo, Norway; 5 Frambu National Resource Center for Rare Disorders, Siggerud, Norway; 6 NORMENT, Institute of Clinical Medicine, University of Oslo, Oslo, Norway

**Keywords:** Angelman syndrome, Autism spectrum disorder, Epilepsy, Epileptic encephalopathy, Seizure onset

## Abstract

**Background:**

Autism spectrum disorder and epilepsy often co-occur; however, the extent to which the association between autism symptoms and epilepsy is due to shared aetiology or to the direct effects of seizures is a topic of ongoing debate. Angelman syndrome (AS) is presented as a suitable disease model to explore this association.

**Methods:**

Data from medical records and questionnaires were used to examine the association between age of epilepsy onset, autism symptoms, genetic aberration and communication level. Forty-eight participants had genetically verified AS (median age 14.5 years; range 1–57 years). A measure of autism symptoms (the Social Communication Questionnaire; SCQ) was completed for 38 individuals aged ≥ 4 years. Genetic cause was subgrouped into deletion and other genetic aberrations of the 15q11-q13 area. The number of signs used to communicate (< 20 sign and ≥ 20 signs) was used as a measure of nonverbal communication.

**Results:**

Mean age of epilepsy onset was 3.0 years (range 3 months–7.8 years). Mean SCQ score for individuals without epilepsy was 13.6 (SD = 6.7) and with epilepsy 17.0 (SD = 5.6; *p* = 0.17); 58% used fewer than 20 signs to communicate. There were no age differences between groups according to presence of epilepsy, level of nonverbal communication or type of genetic aberration. SCQ scores were higher in individuals with the deletion than in those with other genetic aberrations (18.7 vs 10.8 *p* = 0.008) and higher in the group who used < 20 signs to communicate (19.4 vs 14.1 *p* = 0.007). Age of epilepsy onset was correlated with SCQ (*r* = − 0.61, *p* < 0.001). Multiple regression showed that age of seizure onset was significantly related to SCQ score (*β* = − 0.90; *p* = 0.006), even when the type of genetic abnormality was controlled (*R*^2^ = 0.53; *F* = 10.7; *p* = 0.001).

**Conclusions:**

The study provides support for the notion that seizures themselves contribute more to autism symptoms than expected from the underlying genetic pathology alone. The study demonstrates how a rare genetic syndrome such as Angelman syndrome may be used to study the relation between epilepsy and autism symptomatology.

## Background

Angelman syndrome (AS) is a neurodevelopmental disorder caused by an absent or non-functioning maternal allele of chromosome 15q11-q13 [1]. The typical AS phenotype is characterized by intellectual disability (ID), lack of speech, hyperactivity, ataxic gait, microcephaly, sleep disturbances, frequent laughter/smiling and an apparently happy demeanour [1–4]. ID ranges from moderate to profound, with most individuals functioning in the severe to profound range [5, 6]. Epilepsy occurs in 80% or more of cases [2, 7], usually involving multiple seizure types and starting in early childhood [7, 8]. High rates of autistic symptoms are also reported [9–11], with prevalence estimates of autism spectrum disorder (ASD) ranging from 24 to 81% [6, 10]. AS can be due to *UBE3A* mutations, uniparental disomy and imprinting defects [1, 12], but deletions are the predominant cause and are found in 68–75% of patients. Deletions are also associated with more severe AS-phenotype, and co-deletion of GABA_A_-receptor genes (*GABRB3*, *GABRA5* and *GABRG3*) located adjacent to *UBE3A* gene is suggested as a possible explanation for this [1]. Dysfunction of *GABRB3* is highly associated with both epilepsy and autism symptoms [13, 14].

A strong association between autism symptoms, epilepsy and ID has been found in a number of other genetic syndromes, such as fragile X and tuberous sclerosis complex (TSC), as well as in AS [6, 10]. It is evident, too, that the negative effect of seizures is particularly strong during infancy and early childhood [15–18]. Thus, onset of seizures during the first year of life is associated with increased prevalence and severity of ID and ASD and increased prevalence of brain abnormalities [19, 20]. However, there is a continuing debate [21–24] as to whether autism symptoms, epilepsy and ID are independent comorbidities [15, 16, 21, 25–27], whether they are all outcomes of the same underlying pathophysiological/genetic mechanisms [17, 21, 25, 28], or whether the epilepsy itself contributes to more severe cognitive and behavioural impairments than might be expected from the underlying pathology alone [15, 17, 29, 30], i.e. a so-called encephalopathic effect [30].

There are several reasons why AS offers a suitable disease model to investigate the association between epilepsy, ID and autism symptoms. Firstly, the rate of epilepsy in AS (> 80%) is as high as or higher than other genetic disorders in which epilepsy and autism commonly co-occur (e.g. TSC [80–90%]; fragile X syndrome [10–20%]) [29, 31, 32]. Secondly, epilepsy in AS tends to start in very early childhood. Seizures are also often treatment-resistant and refractory epilepsy has been shown to be an important predictor of autism symptoms [33]. Thirdly, unlike genetic conditions such as TSC, in which the numbers and location of tubers are associated with autism symptoms [17, 34], there are no specific structural brain abnormalities in AS that are known to affect the phenotype. Fourthly, knowledge of the specific genetic defects that cause AS makes it possible to evaluate the degree to which the association between epilepsy and autism symptoms is a result of the underlying genetic abnormality and to assess the independent contribution of seizures on level of autism symptoms.

The aims of the current study were to describe epilepsy characteristics and then investigate the relationship between epilepsy, autism symptoms, communication level and genetic cause in individuals with AS. Based on previous research on other populations with childhood epilepsy including TSC [18, 33, 35–37], we hypothesized that age of onset of epilepsy would be related to the number of autism symptoms in AS independent of the effect of the specific genetic abnormality.

## Methods

The study was approved by the regional ethics committee in Norway (REK 2014/1880).

### Recruitment procedures

**Fig. 1:**
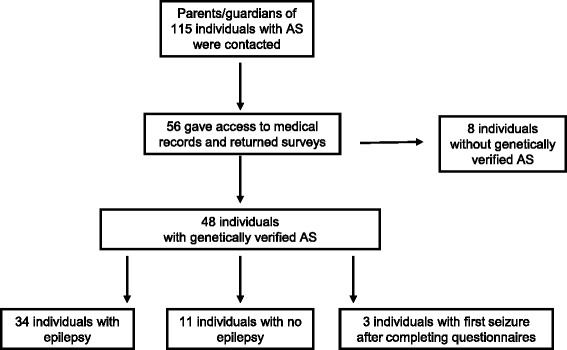
Recruitment

From the records of the Frambu Resource Centre for Rare Disorders in Norway and the Norwegian Angelman Association, 115 individuals with AS were identified. Letters were sent to the parents/guardians of these individuals, and they were asked to complete two questionnaires: the Social Communication Questionnaire (SCQ), which measures autism symptoms [38], and a study-specific questionnaire assessing epilepsy, medication and developmental parameters. Written informed consent was given by all parents/guardians allowing the researchers access to medical records from all hospitals in Norway (Fig. 1).

### Measures

#### Clinical information on epilepsy and genetic abnormality

Participants’ medical records were used to collect information regarding epilepsy and the nature of the genetic abnormality. Information on age of epilepsy onset, type of seizure and treatment with anti-epileptic drugs was recorded when available. Medical records were not comprehensive for all individuals, and formal seizure classification was not always performed.

Genetic data were also variable. When information was available, the genetic abnormality was dichotomised into ‘deletion’ or ‘other’ (i.e. uniparental disomy, imprinting defects and point mutations).

#### Autism symptoms

The lifetime version of SCQ was used to assess the number of autism symptoms [38]. The SCQ contains 40 items scored 0 or 1 and was designed to screen for a possible diagnosis of autism in individuals aged 4 years and older and with a mental age above 2 years [38]. It has also frequently been used to measure autistic-type symptoms in individuals with genetic syndromes including those with AS [9, 11]. We did not classify participants as meeting/not meeting the suggested cut-off scores for autism or ASD (≥ 22 and ≥ 15, respectively [38]) since the validity of these criteria has not been established for individuals with genetic disorders associated with severe ID. Nevertheless, SCQ has often been used as the screening tool in samples with low IQ [39, 40].

#### Communication level

Information about level of development was particularly variable and often very limited. Although many parents reported that they had previously been told their child had severe to profound intellectual disability (in 7 cases, the description was of ‘moderate’ disability), formal test results were rarely recorded, and hence, the validity of these categories was unknown. Although there were no adequate data on IQ/developmental level, we did have data on communication level. Signing was the major mean of communication for most of the participants; the majority had no use of words and no one used more than 20 words. Categorical ratings of ‘use of signs’ (< 20 and 20–100 and > 100) were used to divide individuals into two groups; those using fewer than 20 signs to communicate and those with more than 20 signs.

### Participants

#### Inclusion criteria

For the descriptive part of the study (‘Epilepsy characteristics’), individuals were included if their parents/guardians gave their consent to participation/access to medical records and if their son/daughter had a genetically verified diagnosis of AS. For the second part of the study (‘Relation between epilepsy and autism symptoms, nonverbal communication level and genetic aberration’), individuals were required to be at least 4 years of age (i.e. minimum age for the SCQ).

Parents/guardians of 56 out of the 115 individuals identified from the records (49%) consented to participate; 48 of these individuals (age range 1–57 years; median 14 years 6 months) had a genetically verified AS diagnosis. At the time of questionnaire completion (see Fig. 1), medical records confirmed that 34 individuals had epilepsy and 11 individuals did not. Three boys (aged 1, 1, and 4 years, respectively) subsequently developed seizures; hence, the 4-year-old was included in the no-epilepsy group in the part 2 of the study. SCQ questionnaires were completed for 38 of 40 individuals aged 4 years or older (SCQ was not completed for two participants aged 57 and 40 years). See Table 1 for participants’ characteristics.

**Table 1:** Characteristics of participants with Angelman syndrome in parts 1 and 2 of study

	Age	Gender	Genetics
Part 1: Epilepsy characteristics (n=48)	Range: 1–57 years Mean: 17.1 years Median: 14.5 years	Male: 30 Female: 18	Deletion: 26 (16 males) UPD: 4 (2 males) Imprinting: 3 (1 male) Mutation: 2 (1 male) Unknown: 13 (10 males)
Part 2: Relation between epilepsy and autism symptoms, nonverbal communication level and genetic aberration (n=40)	Range: 1–57 years Mean: 20.2 years Median: 19.1 years	Male: 30 Female: 15	Deletion: 26 (16 males) UPD: 2 (2 males) Imprinting: 2 (1 male) Mutation: 2 (1 male) Unknown: 12 (9 males)

### Statistical analysis

Associations between quantitative measures were analyzed by parametric statistics in SPSS (*t* test, Pearson’s *r)*. Due to small sample size, Mann-Whitney *U* test was used when comparing SCQ in subgroups with/without epilepsy and when comparing SCQ and age of epilepsy onset in subgroups with/without deletion. Fisher’s exact test was used for categorical data. Due to small and unequal sample sizes, Hedges’ *g* was used for effect sizes. Normality of residuals was checked using visual inspection of *P*-*P* plots. Multiple regression analysis was conducted to assess the impact of ‘age at epilepsy onset’and ‘type of genetic aberration’ on SCQ scores. Due to the combination of dichotomous and continuous covariates, we report the standardized coefficients (*β*). To correct for multiple comparisons, a significance level of *p* ≤ 0.01 was chosen; Bonferroni ‘rule of thumb’ was used to determine appropriate *p* level (*p* = 0.05/5 = 0.01).

## Results

### Part 1: epilepsy characteristics

Age of first seizure ranged from 3 months to 7 years 10 months (mean 3 years 0 months, SD 2 years 2 months). Four individuals had their initial seizure during the first year of life; 11 developed epilepsy during the second year. The number and type of seizures varied among individuals and varied over time in the same individuals. Two individuals (aged 38 and 27 years) had been diagnosed with Lennox-Gastaut syndrome. One individual had only ‘atypical absence seizures’, and all others had seizures with ‘jerks’ or ‘convulsions’. More than one seizure type was recorded in 33 individuals. Seizures resembling generalized tonic-clonic seizures (sometimes described as generalized convulsions) were reported in 29 individuals. Seizures resembling atypical absence seizures were seen in 17 individuals, myoclonic seizures in 10 and atonic seizures in 13. Focal seizures were seen in four individuals. Sixteen individuals had their first seizure during a febrile episode, and 10 participants were reported to have epileptic seizures that were aggravated by fever. EEGs were recorded repeatedly in several participants, and findings were typical of those reported in AS [2]. When EEGs were recorded prior to first seizure, delta waves but no epileptiform activity were often reported. More epileptiform discharges in EEGs were recorded during periods of seizure aggravation. Seizures were commonly reported to be resistant to anti-epileptic drugs and drug resistance was particularly marked before 6 years of age, and 21 individuals had received benzodiazepine as emergency treatment. Three individuals had been treated with only one anti-epileptic drug, and all others had tried two or more anti-epileptic drugs. Valproate was the most frequently prescribed anti-epileptic drug (31 participants), followed by nitrazepam (18) and clonazepam (16).

### Part 2: the relation between epilepsy and autism symptoms, nonverbal communication level and genetic aberration

Mean SCQ was 16.3 (SD = 5.9 range: 0–27). SCQ scores were higher in individuals with epilepsy (*n* = 31) than in those without (*n* = 7), but the difference was not significant (see Table 2). SCQ and age were not correlated (*p* = 0.12). Level of nonverbal communication did not differ between individuals with and without epilepsy; 19 of 33 (58%) with epilepsy and 4 of 7 (57%) (exact *p* = 1.000) without epilepsy used fewer than 20 signs to communicate. Individuals with the deletion were more likely to be in the group using < 20 signs to communicate than individuals with other genetic aberrations (exact *p* = 0.022).

**Table 2:** SCQ scores and age at onset of epilepsy according to communication level and genetic aberration

	Epilepsy (*N* = 31)	No epilepsy (*N* = 7)		Epilepsy and level of nonverbal communication (*n* = 31)			Epilepsy and type of genetic aberration (*n* = 23)		
			*p* value [Hedges’ *g*]	< 20 signs (*n* = 17)	≥ 20 signs (*n* = 14)	*p* value [Hedges’ *g*]	Deletion (*n* = 18)	Other genetic aberration (*n* = 5)	*p* value [Hedges’ *g*]
SCQ mean (SD)	17.0 (5.6)	13.6 (6.7)	0.354 [0.59]	19.4 (4.4)	14.1 (5.7)	0.007 [1.05]	18.7 (5.0)	10.8 (6.6)	0.007 [1.48]
Age at onset of epilepsy mean (SD)	36.6 months (26.0)	na	na	30.2 months (18.5)	44.4 months (32.1)	0.160 [0.56]	25.2 months (13.7)	79.0 months (19.3)	< 0.001 [3.60]
Age (SD)	20.0 years (9.7)	13.0 years (9.6)	0.170 [0.72]	21.4 years (10.6)	18.2 years (8.6)	0.57 [0.33]	16.6 years (5.2)	17.4 years (9.5)	0.104 [0.13]

*SCQ* Social Communication Questionnaire score, *na* not applicable

Within the epilepsy group, age of epilepsy onset was lower among individuals using < 20 signs to communicate. Individuals with the deletion had significantly higher SCQ scores and lower age at epilepsy onset than individuals with other genetic aberrations. There were no differences in age between groups (see Table 2 for details).

**Fig. 2:**
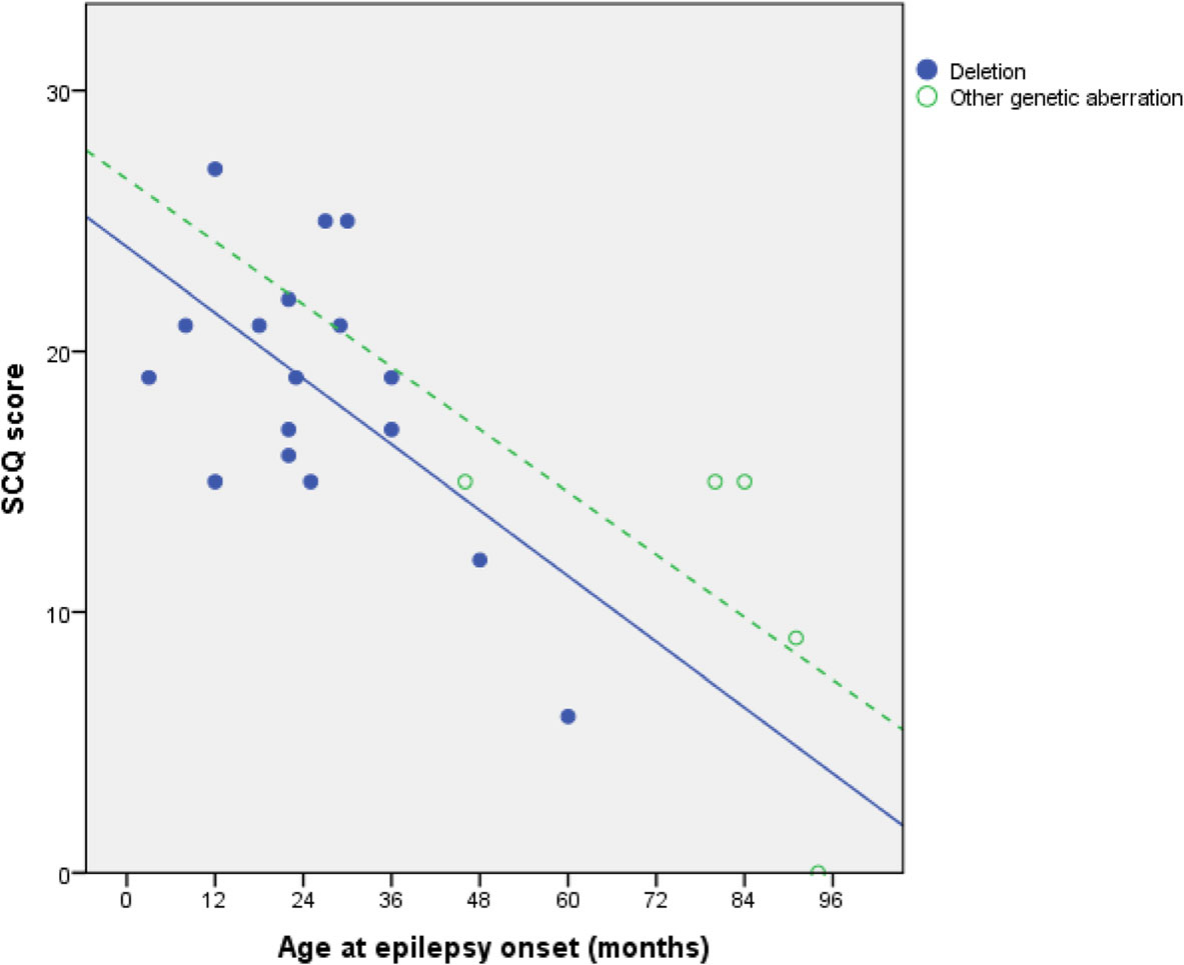
Scatterplot of age at onset of epilepsy and SCQ. Fit-lines are shown according to the type of genetic abnormality

Age at epilepsy onset was highly correlated with SCQ score (*r* = − 0.61, *p* = 0.0004). A linear regression was conducted with SCQ as the dependent variable and age at seizure onset and type of genetic abnormality as the covariates (forced entry). Age at onset of seizures had an independent contribution when entering the type of genetic aberration as a covariate. The type of genetic aberration did not have an independent contribution in this model (see Table 3 and Fig. 2). As a supplementary analysis, we included level of nonverbal communication as a third covariate. Age of epilepsy onset was significant also in this model (*β =* − 0.81, *p =* 0.007).

**Table 3:** Statistical results of regression model with SCQ as dependent outcome

	Standardized coefficients		Unstandardized coefficients		
Covariate	*β*	*p*	*B*	Standard error	95% confidence interval
Model with two covariates *R*^*2*^ = 0.53; *F* = 10, 7; *p* = 0.001					
Age at epilepsy onset	− 0.90	0.006	− 0.21	0.07	− 0.35/− 0.07
Genetic aberration	− 0.22	0.45	− 3.24	4.23	− 12.01/5.61

## Discussion

This study explored the relationship between age of epilepsy onset, autism symptomatology, type of genetic aberration and nonverbal communication level in a Norwegian sample of individuals with AS. Among the 56 individuals with AS identified from the available databases, 48 (86%) had genetically verified AS. This is in line with other reports noting that no genetic abnormality can be identified in 10–15% of individuals with AS [4]. Other clinical findings were similar to those of previous studies of AS. Thus, deletions were the most common genetic cause identified [1, 4]. With regard to epilepsy, the prevalence in this study was 77%, somewhat lower than the rates of ≥ 80% commonly reported [4, 7, 8, 41]. However, our sample included several very young participants who may not yet have had their first seizure. We also excluded individuals in whom the cause of AS was unknown and there is some indication that individuals with AS of unknown cause may have the highest prevalence of seizures [7]. Epilepsy characteristics with early-onset epilepsy, multiple seizure types, a tendency to have seizures during febrile episodes and commonly treatment-resistant seizures, particularly in early childhood, are also in line with the findings reported by others [2, 7, 8, 41, 42], and the use of anti-epileptic drugs is comparable to other studies [7, 8, 41].

The main focus of the study was the association between age of epilepsy onset and extent of autism symptomatology when type of genetic abnormality was controlled for. Our findings from this study of individuals with AS provide support for the notion that seizures themselves contribute more to autism symptoms than might be expected from the underlying pathology alone [15–17, 21]. As anticipated, individuals with a deletion of 15q11-q13 had substantially more autism symptoms than individuals with other genetic aberrations (*g* = 1.48). However, when entered into a regression model with epilepsy onset, genetic aberration made no significant contribution to the number of autism symptoms reported. Although the lack of an independent effect of type of genetic aberration is likely due to the low number of causes other than deletion, it should be noted that the slope of the regression lines is similar for both genetic subgroups, thus supporting the importance of age at seizure onset across the sample. These findings from AS parallel evidence from studies in other rare disorders such as TSC; although both early seizures and encephalopathy are highly associated with type of genetic abnormality, early seizures may contribute to a worsening of developmental outcome [17, 43]. Similarly, from fragile X syndrome, research indicates that males with the FMR1 premutation are more likely to have ASD and ID if seizures occur in childhood [29, 44].

Although individuals with epilepsy had more autism symptoms than those without epilepsy, and despite a moderate to large effect size, this difference was not significant [15]. This may be due to the rarity of non-epilepsy cases among individuals with AS and hence the very small size of the no-epilepsy group. However, the findings also point towards the importance of viewing epilepsy as a spectrum disorder rather than a dichotomy [15]. Hence, the comorbidity between autism symptoms and epilepsy may be related both to the underlying pathology and to the effect of seizures. The high risk of ASD in populations with early-onset epilepsy has been used to support the encephalopathy hypothesis, i.e. that seizures may cause ASD [16, 25]. Others have argued against this because the relationship is bi-directional and individuals with ASD are at increased risk of future epilepsy and seizures may occur in adolescence or adulthood [21, 22, 45, 46]. This study highlights the importance of considering the additive effects of the underlying genetic aetiology and seizures contributing to autism symptoms in AS, which may be relevant also for other conditions [15, 29]. The encephalopathic effect may be greater when seizures start early. Early-life seizures may result in molecular changes which impact neural network structure, and the hippocampal region may be of particular importance. Molecular changes may also influence the expression of genes involved in autism symptoms and genetic syndromes such as *GABRB3*, *FMR1*, *TSC1* and *TSC2* [16, 29]. Moreover, research suggests that effects of seizures on GABA_A_-receptor expression are age-dependent, a finding that further supports the notion that early seizures are particularly harmful [16].

There was no difference in the level of nonverbal communication between the epilepsy group and no–epilepsy group. Age of first seizure however, was associated with nonverbal communication (*g* = 0.56) and individuals with the lowest level of nonverbal communication had earlier seizure onset than those who used more signs to communicate. A number of other studies has found that earlier age of seizure onset is associated with poorer cognitive outcome [18, 33, 35–37, 47, 48]. Our study did not include a measure of development, only a measure of nonverbal communication. However, supplemental analysis showed that age of epilepsy remained significant also when nonverbal communication was entered as a covariate. This suggests that the number of autism symptoms was not explained only by the level of nonverbal communication.

Although the findings of this exploratory study have potentially important implications for understanding the complex links between autism symptoms and epilepsy, there are a number of limitations that must be taken into account in the interpretation of the data. Firstly, the sample size was small and the age of participants was very wide, ranging from infancy to adulthood. In addition, we did not have data on the level of ID, only an estimate of nonverbal communication was available. There were also few individuals with a genetic cause other than the 15q11 deletion, and we lacked data on size of deletions. Furthermore, information from medical records was often incomplete and formal seizure classification, except for tonic-clonic seizures, was rarely performed. Hence, some individuals may have had more types and higher frequency of seizures than reported (particularly those of short duration or less severe such as absences and myoclonic seizures). Finally, there was no clinical assessment of autism, and rather than a categorical distinction between ASD/non-ASD, we focused on the frequency of autism symptoms as measured by the SCQ. While this avoided the problems of misdiagnosing ASD in a population with severe developmental delay, it is well established that the number of autism symptoms is highly related to severity of ID [11]. Thus, high rates of autism symptoms were to be expected in this sample of individuals with AS [9, 10]. The severity of ID in AS is the main limitation when using this disorder as a disease model for studying the relation between autism symptoms and epilepsy.

It is clear that information from a larger sample of individuals with AS, with a larger range of genetic causes other than deletions, and detailed information on developmental level is needed to increase confidence in the current findings. More details of the genetic aberration, such as size and exact break points of the deletions, are also needed. Finally, further studies in this area should investigate which autism symptoms are particularly vulnerable to early seizures and which are less affected. Such knowledge may be of relevance for better understanding of the biology of ASD.

## Conclusions

This study provides support for the notion that, in individuals with AS, seizures themselves contribute more to autism symptoms than expected from the underlying genetic pathology. This study demonstrates how a rare condition may illuminate core issues in research on developmental disorders. Individuals with Angelman syndrome show limited variation in genetic aetiology, and the condition is therefore a suitable one in which to investigate the relation between epilepsy and autism symptoms.

## Abbreviations

AS: Angelman syndrome
ASD: Autism spectrum disorder
ID: Intellectual disability
SCQ: Social Communication Questionnaire
TSC: Tuberous sclerosis complex
